# Control of proton transport and hydrogenation in double-gated graphene

**DOI:** 10.1038/s41586-024-07435-8

**Published:** 2024-06-19

**Authors:** J. Tong, Y. Fu, D. Domaretskiy, F. Della Pia, P. Dagar, L. Powell, D. Bahamon, S. Huang, B. Xin, R. N. Costa Filho, L. F. Vega, I. V. Grigorieva, F. M. Peeters, A. Michaelides, M. Lozada-Hidalgo

**Affiliations:** 1 Department of Physics and Astronomy, University of Manchester, Manchester, UK; 2 National Graphene Institute, University of Manchester, Manchester, UK; 3 Yusuf Hamied Department of Chemistry, University of Cambridge, Cambridge, UK; 4 Research and Innovation Center on CO2 and Hydrogen (RICH Center) and Chemical Engineering Department, Khalifa University, Abu Dhabi, United Arab Emirates; 5 Research and Innovation Center for Graphene and 2D materials (RIC2D), Khalifa University, Abu Dhabi, United Arab Emirates; 6 Departamento de Física, Universidade Federal do Ceará, Fortaleza, Brazil; 7 Departement Fysica, Universiteit Antwerpen, Antwerp, Belgium

**Keywords:** Chemical physics, Electronic properties and devices

## Abstract

The basal plane of graphene can function as a selective barrier that is permeable to protons^1,2^ but impermeable to all ions^3,4^ and gases^5,6^, stimulating its use in applications such as membranes^1,2,7,8^, catalysis^9,10^ and isotope separation^11,12^. Protons can chemically adsorb on graphene and hydrogenate it^13,14^, inducing a conductor–insulator transition that has been explored intensively in graphene electronic devices^13–17^. However, both processes face energy barriers^1,12,18^ and various strategies have been proposed to accelerate proton transport, for example by introducing vacancies^4,7,8^, incorporating catalytic metals^1,19^ or chemically functionalizing the lattice^18,20^. But these techniques can compromise other properties, such as ion selectivity^21,22^ or mechanical stability^23^. Here we show that independent control of the electric field, *E*, at around 1 V nm^−1^, and charge-carrier density, *n*, at around 1 × 10^14^ cm^−2^, in double-gated graphene allows the decoupling of proton transport from lattice hydrogenation and can thereby accelerate proton transport such that it approaches the limiting electrolyte current for our devices. Proton transport and hydrogenation can be driven selectively with precision and robustness, enabling proton-based logic and memory graphene devices that have on–off ratios spanning orders of magnitude. Our results show that field effects can accelerate and decouple electrochemical processes in double-gated 2D crystals and demonstrate the possibility of mapping such processes as a function of *E* and *n*, which is a new technique for the study of 2D electrode–electrolyte interfaces.

## Abstract (web summary)

Independent control of the electric field and charge-carrier density in double-gated graphene allows the decoupling of proton transport and lattice hydrogenation, enabling both accelerated proton transport and proton-based logic operations.

## Main

The charge density, *n*, and the electric field perpendicular to an electrode–electrolyte interface, *E*, are fundamentally linked through applied electrical potential and experimental conditions such as ion concentration or solvent polarizability^24^. By contrast, electron-transport studies have established that electrostatically gating a 2D crystal on both of its surfaces, a technique known as double gating, by using either crystalline dielectrics^25–28^ or liquid electrolytes^29–31^, enables the decoupling of *E* and *n* because the individual gate potentials superpose in the 2D crystal. The independent control of these variables in 2D electronic transport devices^25–30^ is now being routinely used to modify the band structure of 2D crystals, for example to quench the bandgap of 2D semiconductors^29,30^ or to enable precise electrostatic control of phases such as coupled ferroelectricity and superconductivity^26^. In this context, we hypothesize that double-gating could enable the study of proton transport^1,2^ and hydrogenation^13,14^ in graphene with independent control of *E* and *n*, which is not currently possible. Pristine graphene, which is impermeable to all atoms and molecules at ambient conditions^5,6^, is permeable to thermal protons in the direction perpendicular to its basal plane^1,2^. It has been suggested that pinholes in the lattice were needed for the transport. However, recent work conclusively showed that the pristine lattice is permeable to protons^2^, and that strain and curvature in wrinkles and nano-ripples intrinsic to the crystal lower the energy barrier for the transport. Conversely, the hydrogenation of graphene^13,15^, which was originally studied using hydrogen plasmas^13,32,33^, has been shown to proceed efficiently in an electrochemical set-up using a non-aqueous electrolyte^14^. This process is characterized by a reversible but hysteretic gate-controlled conductor–insulator transition in graphene^14^, accompanied by a prominent D band in its Raman spectrum. In this work, we study these two well-known electrochemical processes with independent control of *E* and *n* in graphene and find that this enables these processes to be driven with otherwise unattainable selectivity.

## Double-gated graphene devices

**Fig. 1:**
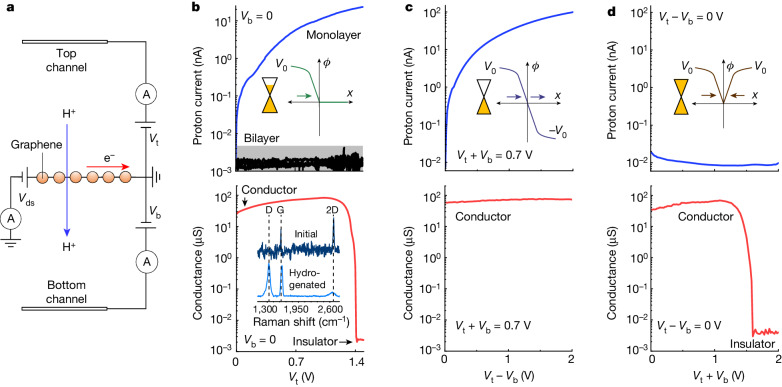
Selective control of proton transport and hydrogenation in double-gated graphene devices. **a**, Schematic of devices used in this work. A, ammeter. **b**, Proton transport and hydrogenation. Top, proton current–voltage characteristics from monolayer graphene devices for *V*_b_ = 0 (blue curve). In this gate configuration, *E* and *n* are coupled. Black curve, corresponding characteristics for bilayer graphene. Grey area, resolution background determined by parasitic leakage currents. Bottom, corresponding in-plane conductance, demonstrating a conductor–insulator transition. *V*_ds_ = 0.5 mV. Bottom inset, Raman spectra collected in the conductor (dark blue) and insulator (light blue, displaying a sharp D-band) regime. The background signal from the electrolyte was subtracted from the spectra; the spectrum of hydrogenated graphene was divided by 5 for clarity. **c**, Proton transport, no hydrogenation. Top, proton current in devices as a function of *V*_t_ − *V*_b_ for constant *V*_t_ + *V*_b_ = 0.7 V. In this gate configuration, *n* is constant and *E* is variable. Bottom, the corresponding in-plane electronic conductivity showing that hydrogenation does not take place. **d**, No proton transport, with hydrogenation. Top, proton current in devices as a function of *V*_t_ + *V*_b_ for constant *V*_t_ − *V*_b_ = 0, showing negligible proton transport. In this gate configuration, *E* = 0 and *n* is variable. Bottom, corresponding in-plane electronic conductivity showing that graphene becomes hydrogenated. In **b**–**d**, the top insets show schematics of the gate potential (*φ*) versus the distance from the graphene (*x*) for the different gate configurations (**b***, V*_t_ = *V*_0_ and *V*_b_ = 0, green; **c**, *V*_t_ = *V*_0_ and *V*_b_ = −*V*_0_, purple; **d**, *V*_t_ = *V*_b_ = *V*_0_, brown). The gate potentials shift the Fermi level of graphene with respect to the neutrality point (yellow). The horizontal arrows mark the interfacial electric field induced by each of the gates, which add up to yield the total *E* in graphene.

**Extended Data Fig. 1:**
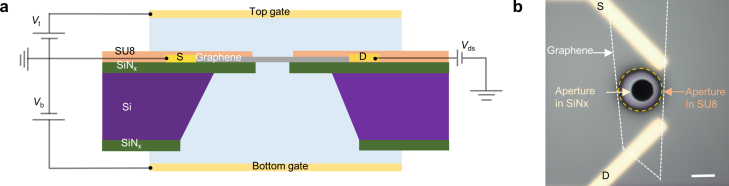
Experimental devices. **a**, Schematic of experimental devices used in this work. **b**, Optical image of devices (top view). Dashed white lines mark the area covered by graphene. Dark circle, aperture in silicon-nitride substrate. Dashed orange circle, aperture in the SU-8 washer. All the area shown in the panel (except for the aperture in SU-8 washer) is covered with the washer. S, D labels mark the source and drain electrodes. Scale bar, 10 μm.

**Extended Data Fig. 2:**
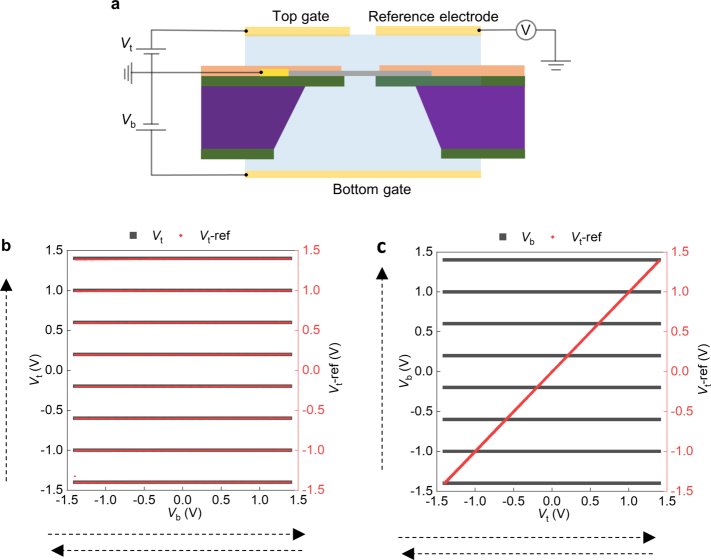
Independence of top and bottom gates. **a**, Schematic of experimental devices with reference electrode. **b**, Voltage measured in the reference electrode, *V*_t_^ref^ (red data points), as a function of *V*_b_ for a fixed *V*_t_ (black data points). The horizontal dashed arrows in the bottom axis indicate that the bottom gate is swept from −1.4 V to and from 1.4 V repeatedly for a fixed value of *V*_t_. Vertical dashed line next to the left *y*-axis indicates that *V*_t_ is stepped from −1.4 V to 1.4 V. Left *y*-axis (black), applied *V*_t_. Right *y*-axis (red), measured *V*_t_^ref^. **c**, Voltage measured in the reference electrode, *V*_t_^ref^ (red data points), as a function of *V*_t_ for a fixed *V*_b_ (black data points). The horizontal and vertical lines next to *x*- and *y*-axis indicate the sweeping and stepping of the gates. Left *y*-axis (black), applied *V*_b_. Right *y*-axis (red), measured *V*_t_^ref^.

**Extended Data Fig. 3:**
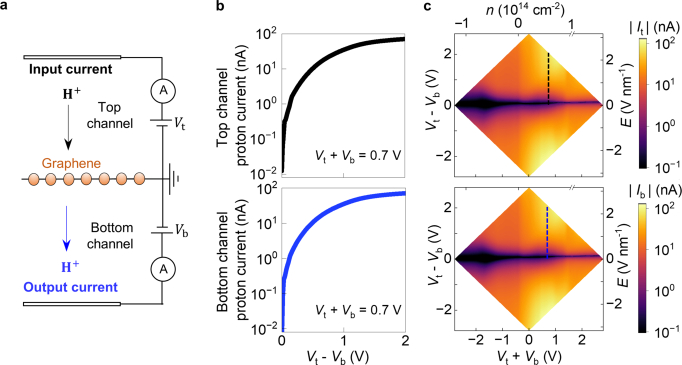
Top and bottom proton channels are symmetrical to each other. **a**, Schematic of devices illustrating the top and bottom channel current. **b**, Top and bottom channel proton transport current taken from the black and blue cross sections in the maps in panel **c** (*V*_t_ + *V*_b_ = 0.7 V), respectively. **c**, Maps of top (top panel) and bottom channel (bottom panel) current as a function of *V*_t_ + *V*_b_ and *V*_t_ – *V*_b_.

Our device configuration consisted of mechanically exfoliated graphene suspended over a small hole (10 μm in diameter) that was etched into silicon nitride substrates, as previously reported^1^ (Methods and Extended Data Fig. 1). The resulting suspended films were coated on both sides with a non-aqueous proton-conducting electrolyte with a large (greater than 4 V) electrochemical-stability window (bis(trifluoromethane)sulfonimide (HTFSI) dissolved in poly(ethylene glycol)^14^) and contacted with two proton-injecting electrodes (PdH_*x*_). For reference, we also measured devices using electrolytes in which free protons were exchanged for Li^+^ ions (substituting HTFSI for LiTFSI). The graphene films were then connected in the electrical circuit shown schematically in Fig. 1a. Two sets of (gate) voltages, *V*_t_ and *V*_b_, were applied between graphene and each of the PdH_*x*_ electrodes, enabling us to control the potential on each graphene–electrolyte interface independently (Extended Data Fig. 2). The applied gate voltages were used to drive the proton transport current in the device (Methods and Extended Data Fig. 3), which is the first process we investigated here. The second process was hydrogenation. To measure the conductor–insulator transition induced by this process, we measured the in-plane electronic conductivity of graphene (applying a drain-source voltage, *V*_ds_) as a function of the applied gate voltages. This set-up enabled the simultaneous measurement of the out-of-plane proton and the in-plane electronic conductivity of graphene.

## Field-effect-enabled selectivity

**Extended Data Fig. 4:**
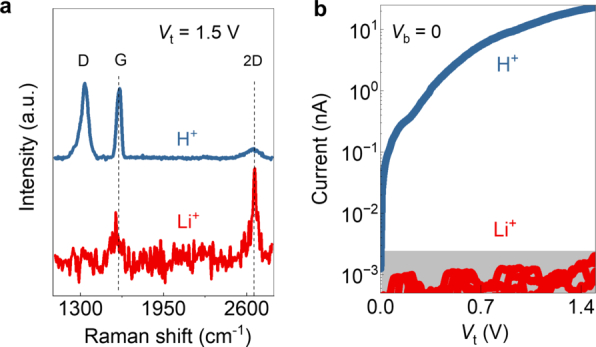
Reference experiments with Li^+^ conducting electrolyte. **a**, Raman spectra for monolayer graphene devices in which free protons are exchanged for Li^+^ ions do not display a *D* band even at high applied gate voltage, demonstrating that the adsorbed species are indeed protons. The background signals from the electrolytes were subtracted and the spectrum of hydrogenated graphene was divided by a factor of 5 for clarity. **b**, Monolayer graphene is completely impermeable to Li^+^. Grey area, experimental resolution background.

**Extended Data Fig. 5:**
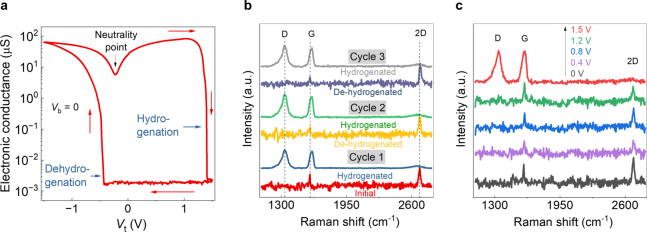
Reversibility of hydrogenation of graphene. **a**, In-plane electronic conductance in graphene as a function of gate voltage *V*_t_ for *V*_b_ = 0. The electronic system undergoes a reversible conducting-insulating transition as *V*_t_ is swept along the loop marked with red arrows. Blue arrows mark the points at which the system undergoes the reversible insulating (hydrogenation) and conductive (dehydrogenation) transitions. The neutrality point is indicated with a black arrow. Drain-source bias, 0.5 mV. **b**, Raman spectra show that the conducting-insulation transition is accompanied by a sharp *D* band, consistent with hydrogenation of the lattice. The devices can be hydrogenated and dehydrogenated multiple times. Dashed lines mark the position of the *D*, *G* and 2*D* bands. **c**, Raman spectra as a function of *V*_t_ show that the *D* band appears suddenly for gate voltages between 1.2 V–1.5 V. The background signal from the electrolyte was subtracted and the spectra of hydrogenated graphene in panels **b** and **c** were divided by a factor of 5 for clarity.

To appreciate the advantages of using two gates, we first characterized proton transport and hydrogenation for the case in which one of the gates (the bottom one) was set to zero, *V*_b_ = 0. The top panel in Fig. 1b (blue curve) shows that applying a bias to the top gate led to proton transport through graphene (*V*_t_ > 0, *V*_b_ = 0). Reference devices fabricated with bilayer graphene, which is impermeable to protons^1^, displayed no current within our experimental resolution (black curve in Fig. 1b). Moreover, monolayer graphene devices measured with Li^+^-conducting electrolyte displayed no current either (Extended Data Fig. 4), which is consistent with the known impermeability of graphene to all ions^3,4^. These experiments confirm that the transport current observed in monolayer devices is indeed due to proton transport. However, the bottom panel in Fig. 1b shows that when we drive proton transport through monolayer graphene, the in-plane electronic conductivity drops by four orders of magnitude around *V*_t_ ≈ 1.4 V, turning graphene into an electronic insulator, and that a prominent D band appears in the Raman spectrum of graphene (Fig. 1b, bottom inset and Extended Data Fig. 5). These results therefore show that if one of the gates is set to zero, accelerating proton transport with the other gate eventually leads to hydrogenation of the lattice. Unexpectedly, we found that using two gates allowed us to drive strong proton transport through graphene without hydrogenation. The response of the devices when the gate voltages were set such that their sum was fixed, *V*_t_ + *V*_b_ = 0.7 V, but their difference was variable, *V*_t_ − *V*_b_ > 0, is shown in Fig. 1c. This yielded strong proton transport current (top) but, crucially, graphene remained electrically conductive even for large gate potentials, demonstrating that hydrogenation did not take place (bottom). The converse is also possible. Setting the gate potentials such that their difference was fixed, *V*_t_ – *V*_b_ = 0, but their sum was variable *V*_t_ + *V*_b_ > 0, could suppress proton transport (Fig. 1d, top), but graphene became hydrogenated (bottom). These results demonstrate that double-gated devices allowed us to drive the two processes selectively, even at high bias, which is not possible using only one gate.

**Extended Data Fig. 6:**
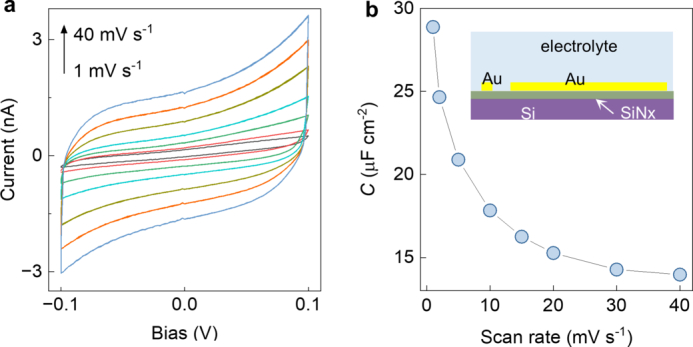
Characterization of electrolyte capacitance. **a**, Cyclic voltammetry characteristics of reference devices in which HTFSI electrolyte is in contact with two mm-sized Au electrodes (inset panel **b**). The different curves (colour coded) were obtained at sweep rates ranging from 1 mV s^−1^ (black) to 40 mV s^−1^ (dark blue). **b**, Geometrical capacitance per unit area as a function of sweep rate extracted from the CV curves shown in panel **a**. Inset, schematic of reference devices used for this experiment. The dimensions of the active area of the electrodes were 4 mm × 4 mm and 0.3 mm × 1 mm.

To understand why double gating enables this decoupling, we recall previous research^25–30^ that showed that, in double-gated 2D crystals, *E* ∝ *V*_t_ – *V*_b_, whereas *n* depends on only *V*_t_ + *V*_b_. This point is discussed quantitatively in the Methods but can be understood qualitatively as follows. Consider the case in which both gates are fixed at the same potential (*V*_t_ = *V*_b_ = *V*_0_), illustrated in the inset in Fig. 1d. Both gates shift the Fermi level (*μ*_e_) of graphene in the same direction, and because *n* ∝ *μ*_e_^2^, this raises *n*. However, the electric fields in the two graphene–electrolyte interfaces, shown by the gradients of the gate potentials (Fig. 1d inset, horizontal arrows), point in opposite directions, so they yield zero total *E* in the 2D crystal. In this case then, *V*_t_ + *V*_b_ = 2*V*_0_ and *V*_t_ – *V*_b_ = 0, which illustrates that *n* and *E* are determined by the sum, and the difference of the gate potentials, respectively. Conversely, if the gates have opposite polarity (*V*_b_ = –*V*_0_ = –*V*_t_; Fig. 1c inset), *μ*_e_ is driven in opposite directions by the gates. This yields zero induced *n* but the electric fields induced by each gate now point in the same direction, yielding high *E* in graphene (*V*_t_ + *V*_b_ = 0, *V*_t_ – *V*_b_ = 2*V*_0_). The solution to the corresponding electrostatic equations shows that *E* and *n* are indeed functions of *V*_t_ – *V*_b_ and *V*_t_ + *V*_b_, respectively, and depend only on the capacitance of the electrolyte and fundamental constants (Methods). Direct characterization of such capacitance (*C* ≈ 20 µF cm^–2^; Extended Data Fig. 6) reveals that our devices can achieve *E* of around 1 V nm^–1^ and *n* of around 10^14^ cm^–2^. This discussion therefore reveals that decoupling proton transport from hydrogenation is possible here because of the independent control of *E* and *n* in double-gated graphene. We explored this decoupling systematically by mapping both processes in terms of these variables.

## Proton and electronic transport maps

**Fig. 2:**
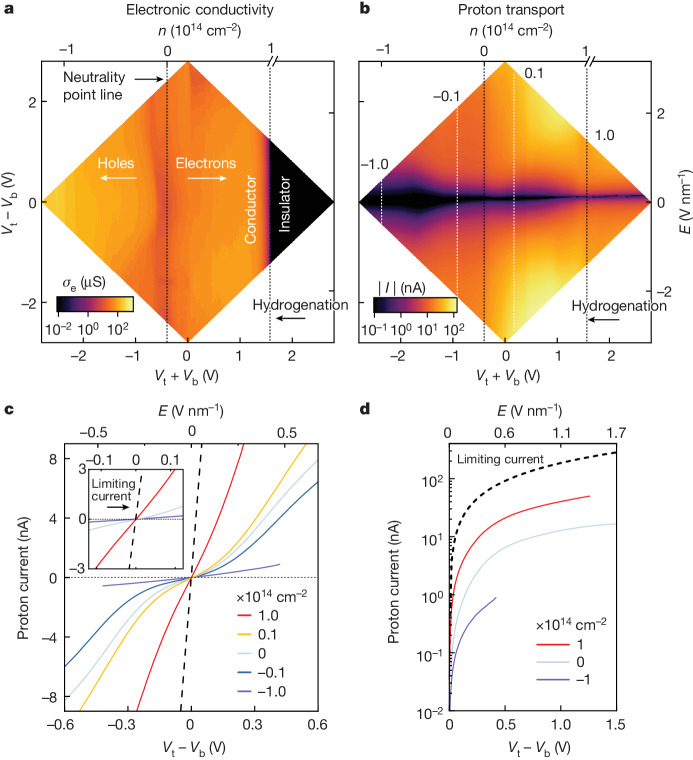
Proton and electronic transport with independent control of *E* and *n* in double-gated graphene. **a**, Map of in-plane electronic conductance, *σ*_e_, as a function of *E* and *n* (the left and bottom axes show that these variables are controlled by *V*_t_ − *V*_b_ and *V*_t_ + *V*_b_, respectively). The top axis (*n*) is cut off after the hydrogenation transition because the scale no longer applies (how *E* and *n* were estimated is described in the Methods). The NP line is visible as a slightly darker band in the map (vertical dashed line). The conductor–insulator transition that results from hydrogenation is shown as a sharp boundary for high electron doping (dashed line). **b**, Map of proton transport current, *I*, as a function of *E* and *n*. Dashed lines mark the cross-sections at constant *n* shown in **a**. The number labels mark the fixed *n* for each cross-section in units of 10^14^ cm^−2^. **c**, Proton transport current as a function of *E* for constant *n*. Each *I*–*E* curve is a cross-section taken from **b**. The number labels mark the fixed *n* for each cross-section in units of 10^14^ cm^−2^. The dashed line marks the limiting current enabled by the electrolyte obtained from devices without graphene (Extended Data Fig. 9). The dotted horizontal line is a guide to the eye. Inset, zoomed in from the main panel for *E* < 0.1 V nm^−1^ in which the transport characteristics are linear. **d**, Proton transport current as a function of *E* for constant *n* in the high-*E* regime. Number labels mark the fixed *n* for each cross-section in units of 10^14^ cm^−2^.

**Extended Data Fig. 7:**
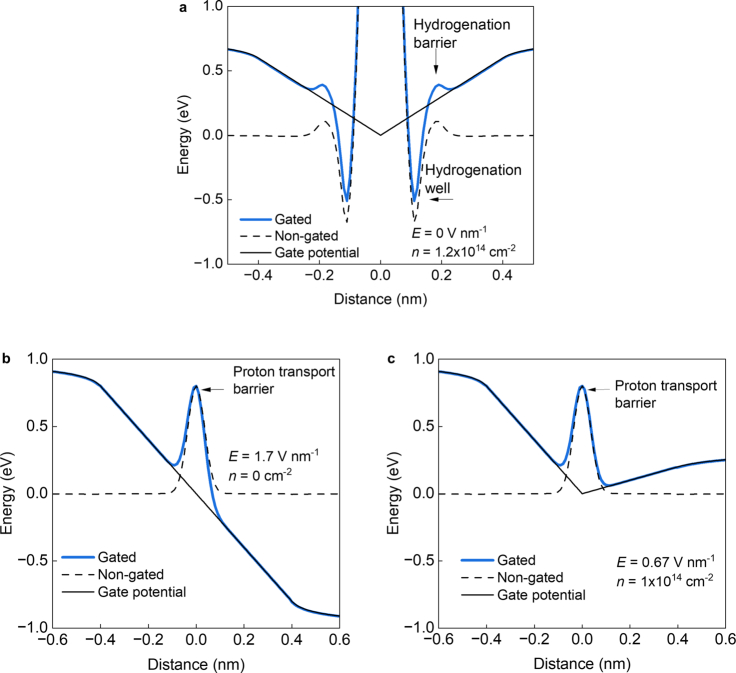
Analytical model of proton transport and hydrogenation in double-gated graphene. **a**, High electron doping effectively removes the energy barrier for hydrogenation. Energy profile for graphene hydrogenation in the absence of a gate (dotted curve), the gate potential (solid black curve) and their superposition (blue curve). *V*_t_ = *V*_b_ = 0.74 V. **b**, High electric field lowers the effective energy barrier for proton transport. Energy profile for proton transport through graphene in the absence of a gate (dotted curve), the gate potential (solid black curve) and their superposition (blue curve). *V*_t_ = −*V*_b_ = 0.96 V. **c**, High electron doping lowers the effective energy barrier for proton transport. Energy profile for proton transport through graphene in the absence of a gate (dotted curve), the gate potential (solid black curve) and their superposition (blue curve). *V*_t_ = 0.96 V, *V*_b_ = 0.29 V.

**Extended Data Fig. 8:**
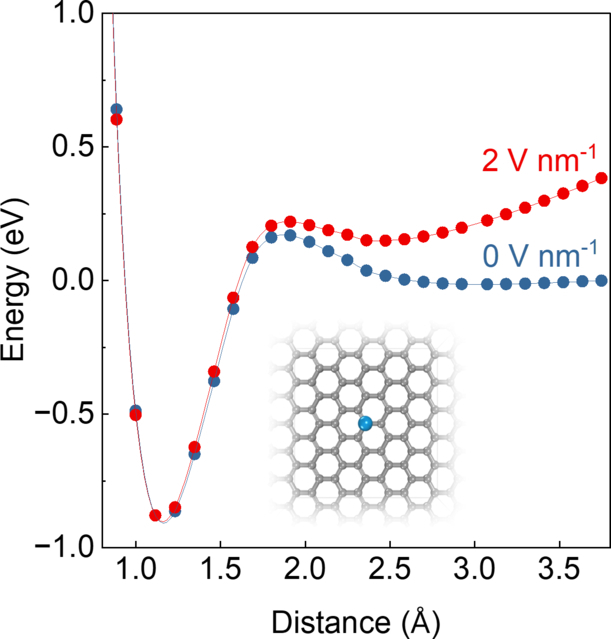
Calculated potential energy profile for graphene hydrogenation. Potential energy versus distance for a proton over a carbon atom in graphene. An electric field perpendicular to the graphene sheet distorts the potential energy profile (red data points), with respect to the case where no field is applied (blue data points). Red and blue curves, spline interpolation to data. Inset, schematic of a proton (blue ball) on top of the graphene lattice (grey balls).

We started with hydrogenation. The in-plane electronic conductivity of graphene was mapped by sweeping *E* for a fixed *n* and then stepping *n* from hole-doped regions towards electron*-*doped ones. As shown in Fig. 2a and Extended Data Fig. 5, the conductivity displayed a local minimum, the charge neutrality point (NP), which was visible as a vertical band at –0.35 ± 0.15 V that split the map between hole-doped and electron-doped regions. To the right of the NP, where graphene was electron-doped, we found a conductor–insulator transition, evident as a sharp boundary at *n* ≈ 1 × 10^14^ cm^–2^, that was accompanied by the sudden appearance of a D band in the Raman spectrum of graphene (Extended Data Fig. 5). This shows that graphene was hydrogenated, and we find that this state was retained unless a negative gate voltage was applied, resulting in a hysteretic dependence of the process on gate voltage (Extended Data Fig. 5). The density of adsorbed hydrogen atoms in hydrogenated samples can be estimated directly from the intensity of the D band in their Raman spectrum as approximately 10^14^ cm^–2^ (Methods), consistent with the electron doping of graphene at the hydrogenation transition. No D band was observed in reference devices measured with a Li^+^-conducting electrolyte at any applied bias (Extended Data Fig. 4), confirming that the observed phenomena are indeed caused by proton adsorption on graphene. The found dependence of the hydrogenation process on *n* can be rationalized using a classical analytical model. This shows that the energy barrier for hydrogenation is effectively suppressed for the potential configuration that led to large electron doping (Extended Data Fig. 7), in agreement with our density functional theory (DFT) calculations (Extended Data Fig. 8 and Methods). The dependence can also be understood by noticing that *n* is related to the electrochemical potential of electrons with respect to the NP in graphene as ${\mu }_{{\rm{e}}}\propto \sqrt{n}$. Such a relation implies that the hydrogenation process was driven by *μ*_e_, which is consistent with the well-established notion that electrochemical charge transfer processes are driven by this potential (Methods).

**Extended Data Fig. 9:**
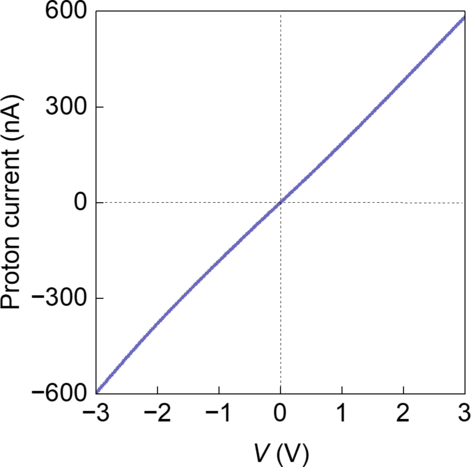
Characterization of reference devices without graphene. **a**, Examples of *I*-*V* characteristics of ‘open hole’ device. The device consists of a 10 μm diameter hole etched in a silicon nitride substrate; HTFSI electrolyte on both sides; and two PdH_x_ electrodes. Dashed lines, guide to the eye.

**Extended Data Fig. 10:**
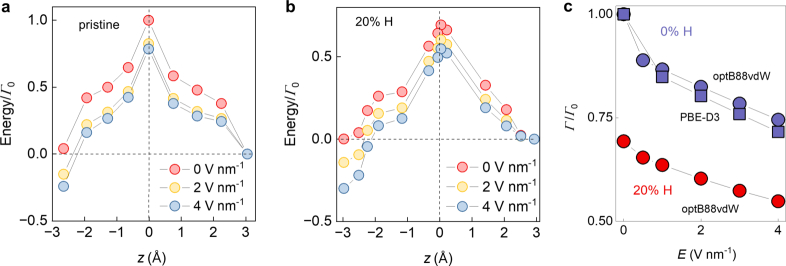
Calculated energy barriers for proton transport through graphene. **a**, Energy barriers for proton transport through graphene under different electric fields for no hydrogenation and **b**, for 20% H atom coverage, normalized versus *Γ*_0_, the barrier height for the case of no H atoms absorption and zero electric field. **c**, DFT calculations show that *E* and H atom adsorption lower the energy barrier for proton transport, *Γ*. Red (blue) symbols, *Γ*/*Γ*_0_ for the case of no H atom adsorption (20% H coverage). Circle and square symbols, data obtained using the optB88-vdW and PBE-D3 functionals, respectively.

We now discuss proton transport. Figure 2b shows the proton transport map obtained simultaneously with the electronic map in Fig. 2a. To analyse it, we took cross-sections of the map at constant *n* from hole-doped and electron-doped regions (dotted lines in Fig. 2b). As shown in Fig. 2c, proton transport was driven by *E*, but the current was notably larger when graphene was electron doped. This finding could be analysed quantitatively in the low-*E* regime (*E* < 0.1 V nm^–1^), in which the proton transport characteristics are linear. The inset in Fig. 2c shows that, for electron doping of around 1 × 10^14^ cm^–2^, graphene was about 30 times more proton conductive than for hole doping with the same concentration and reached values about five times lower than the limiting conductivity enabled by the electrolyte (black dashed line in Fig. 2c). For larger fields, the transport characteristics became nonlinear, and for even larger fields of more than 0.5 V nm^–1^, the transport for all doping regimes traced the limiting current enabled by the electrolyte, but attenuated according to their doping (Fig. 2d). This field effect is reversible and does not arise from changes in the electrolyte conductivity with bias (Extended Data Fig. 9). It is an intrinsic effect of graphene at high *E* and high *n*. The dependence of proton transport on these variables can be understood by using a similar analytical model to the one used to study the hydrogenation transition. The model reveals that configuring the gate potentials to dope graphene with electrons distorted the potential energy profile for the incoming protons, facilitating their transport with respect to the case in which graphene was not doped. Large *E* also distorted this energy profile, providing incoming protons with energy comparable with the energy-barrier height, thereby facilitating the transport for any doping configuration (Extended Data Fig. 7). This model is consistent with our DFT calculations, which show that *E* reduced the energy barrier for proton transport with respect to the zero-field case (Extended Data Fig. 10) and as such should result in a strong acceleration of the transport.

## Precise and robust control of processes

**Fig. 3:**
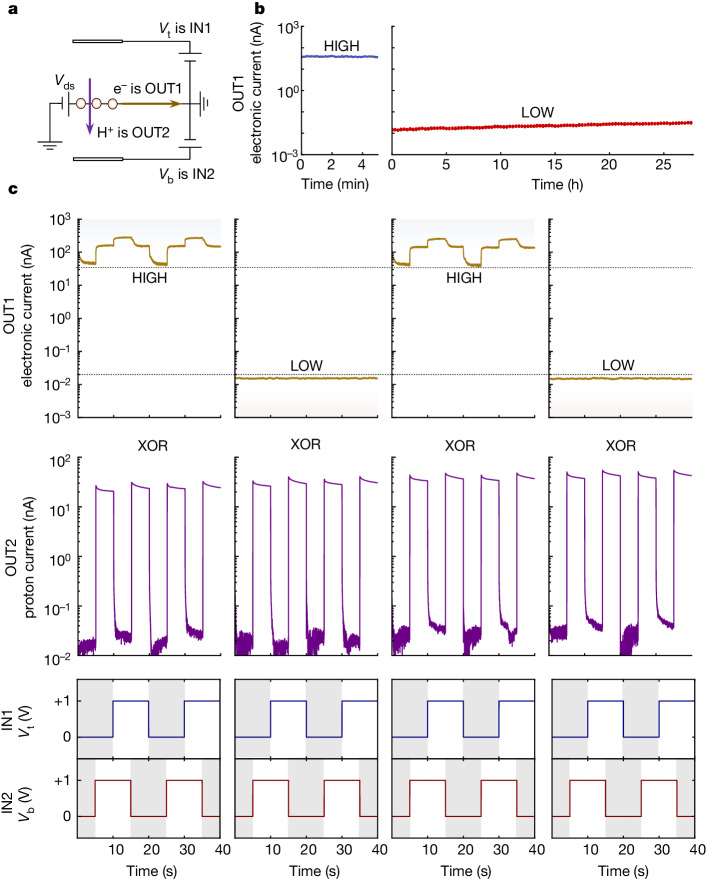
Robust and precise switching of proton transport and hydrogenation in double-gated graphene enables proton-based logic and memory devices. **a**, Schematic of the signal protocol. *V*_t_ and *V*_b_ are set as the logic inputs, IN1 and IN2, respectively. The electronic current is defined as OUT1 and functions as the memory state. The proton current is set as the logic output, OUT2. **b**, Demonstration of long-term memory-state retention. Left, the device is in the HIGH (conductive) state with retention tested under IN1 = IN2 = 0 V. Right, the device is pre-programmed into the LOW (insulating) state with retention that is also tested under IN1 = IN2 = 0 V. The LOW state is programmed by applying 1.4 V on both gates, and the HIGH state is recovered by applying −1.4 V on both gates. **c**, Demonstration of simultaneous logic and memory operations using the out-of-plane proton system as the logic gate and the in-plane electronic system as the memory unit. The bottom two rows show the input signals, IN1 and IN2, which are squared waveforms with various combinations of input values (00, 01, 11, 10, in two cycles). The middle row shows the dynamic response of the proton currents. Under the input levels (00 or 11), OUT2 displays low currents of around 20 pA, whereas for different levels (01 or 10) it displays high currents of around 30 nA, thus demonstrating an XOR logic gate. The top row shows the dynamic response of the electronic current (source-drain bias of 0.5 mV) measured simultaneously with the out-of-plane proton current. The in-plane electronic system of graphene was pre-programmed into the dehydrogenated HIGH memory state and the hydrogenated LOW memory state (marked with dashed lines and shaded areas).

We investigated the precision and robustness with which we can selectively drive the proton transport and hydrogenation processes. To that end, we evaluated the performance of the devices in logic and memory applications (Fig. 3a). The in-plane electronic system was used as a memory unit and the hydrogenation process was used to program two memory states, HIGH (conducting) and LOW (insulating), such that the ratio of their conductivity exceeded 10^3^. As shown in Fig. 3b, the memory states were non-volatile and were retained for more than one day, as long as we decided to measure. This non-volatility is a consequence of the hysteretic dependence of the hydrogenation process on *n* that was discussed above (Extended Data Fig. 5). With the electronic system pre-programmed, we used the out-of-plane proton transport current to perform logic operations. The two gates were used to apply high *E* to drive proton currents and perform logic operations, but with *n* that does not disturb the pre-programmed memory state in the electronic system. As shown in Fig. 3c, the proton-transport system yielded an XOR logic operator with on–off ratios of more than two orders of magnitude, and that during its operation the in-plane electronic memory state remained in its pre-programmed (conducting or insulating) state. This demonstrates that control of the processes is robust and precise enough to enable computation applications. Here, graphene performs both logic and memory functions by means of the independent control of its proton- and electronic-transport properties. This combination of functions in the same physical area of the device would eliminate^34^ the need for peripheral circuits between the logic and memory components in prospective arrays of these devices, making them more energy efficient and compact. Using 3D ensembles that exploit the independent proton- and electron-current pathways could enable even denser proton-based logic and memory networks^35^.

## Outlook

We have shown that double-gated graphene devices enable precise and robust control of proton transport and hydrogenation by means of the independent control of *E* and *n*. We have shown that, in double-gated graphene, the proton current can be used to perform logic operations, which is of interest in the field of electrochemically gated electronic materials^36,37^. We have shown that field effects strongly accelerate proton transport, which is important for applications in proton-conducting membranes^1,2^, catalysis^9,10^ and isotope separation^11,12^. More generally, we have shown that double-gated 2D crystals enable mapping processes in electrode–electrolyte interfaces as a function of *E* and *n*, which cannot currently be achieved without double gating. We call these maps electrochemical charge–field maps to differentiate them from conventional double-gating, in which the gate (electrolytic) current is not monitored as a meaningful variable. The selective acceleration of proton transport and hydrogenation reported here suggests that similar 2D crystal devices could selectively drive other coupled interfacial processes. Given the advances in embedding catalysts in 2D crystals reported previously^38^, such processes might in the future perhaps include reactions such as CO_2_ reduction that could compete with the much faster hydrogen-evolution reaction^39^. Fundamentally, our work expands the parameter space over which electrochemical processes in 2D crystals can be studied.

## Methods

### Device fabrication

Apertures 10 µm in diameter were etched in silicon nitride substrates (500 nm SiN_*x*_) using photolithography, wet etching and reactive ion etching, as previously reported^1^. Source and drain electrodes (Au/Cr) were patterned using photolithography and electron-beam evaporation. Mechanically exfoliated monolayer and bilayer graphene crystals were transferred over the apertures and on the electrodes (Extended Data Fig. 1a). We selected crystal flakes with a rectangular shape, with their long side being several tens of micrometres, to form a conducting channel between the source and drain electrodes. The width of the flake was chosen to be only a couple of micrometres wider than the aperture (Extended Data Fig. 1b), which ensured that a whole cross-section of the flake could be gated with the two gates effectively. The cross-section of the flake became the active area of the device, with the non-gated areas acting as electrical contacts to the gated section. An SU-8 photo-curable epoxy washer with a hole 15 μm in diameter was transferred over the flake and on the source and drain electrodes^3^ with the hole in the washer aligned with the aperture in the silicon nitride substrate (Extended Data Fig. 1b). The polymer seal ensured that the electrodes were electrically insulated from the electrolyte. The electrolyte used was 0.18 M HTFSI dissolved in polyethylene glycol (number-averaged molecular mass, M_n_, of 600)^14^, never exposed to ambient conditions. This electrolyte was drop-cast on both sides of the device in a glovebox containing an inert gas atmosphere. For reference, we measured devices prepared in the same way, except HTFSI was substituted for LiTFSI in the electrolyte. Palladium hydride foils (around 0.5 cm^2^) were used as gate electrodes. The device was placed inside a gas-tight Linkam chamber (HFS600E-PB4) filled with argon for electrical measurements.

### Transport measurements

For electrical measurements, a dual-channel Keithley 2636B sourcemeter was used to bias both gates. The applied voltages yielded two proton current signals, top (*I*_t_) and bottom (*I*_b_) channel current, also recorded with a Keithley sourcemeter. These two signals quantified the two halves of the proton-transport circuit: proton transport from one gate electrode towards graphene, and then from graphene towards the other gate electrode. Because of the proton permeability of graphene, these two currents were effectively identical (Extended Data Fig. 3), differing by only about 1%. For this reason, it is sufficient to use only one of them to unambiguously characterize proton transport in the device, *I*. A second Keithley 2614 A sourcemeter was used both to apply drain-source bias (*V*_ds_) and to measure the electronic conductance (*σ*_e_).

To confirm that the two gates are independent, we connected the electrolyte in the top channel with a reference electrode (PdHx foil, the same size as the gate electrodes), and monitored its potential, *V*_t_^ref^, with a Keithley 2182 A nanovoltmeter (Extended Data Fig. 2a). In the first experiment, we swept *V*_b_ for various fixed *V*_t_. Extended Data Fig. 2b shows that *V*_t_^ref^ = *V*_t_ for all values of *V*_b_ within the experimental scatter of less than 4 mV. This demonstrates that sweeping *V*_b_ does not affect *V*_t_. In the second experiment, we swept *V*_t_ for various fixed *V*_b_. This measurement also showed that *V*_t_^ref^ = *V*_t_ (Extended Data Fig. 2c), which demonstrates that a fixed *V*_b_ does not affect *V*_t_ either. These experiments therefore demonstrate that the gates are independent from each other.

To obtain maps of the proton and electronic systems, we measured *I* and *σ*_e_ simultaneously as a function of *V*_t_ and *V*_b_. The maps were obtained using software that allowed us to control *V*_t_ − *V*_b_ and *V*_t_ + *V*_b_ as independent variables. We swept *V*_t_ − *V*_b_ (for a fixed *V*_t_ + *V*_b_) at a rate of 10 mV s^−1^ for each gate and stepped *V*_t_ + *V*_b_ with intervals of 10 mV. The maximum *V*_t_ or *V*_b_ applied was ±1.4 V, which resulted in a maximum *V*_t_ + *V*_b_ and *V*_t_ − *V*_b_ of ±2.8 V. We normally did not apply *V* bias beyond these voltage ranges to avoid damaging the devices.

### Raman spectroscopy

For Raman measurements, the graphene devices were left in the same gas-tight Linkam chamber (HFS600E-PB4) used for electrical measurements. It has an optical window. The Raman spectra of devices were measured as a function of applied *V* bias using a 514 nm laser. The background signal from the electrolyte was removed for clarity, resulting in relatively weak Raman spectra for pristine graphene (Extended Data Fig. 5). After hydrogenation, a strong *D* peak appeared and the intensity of the *G* peak increased while the 2D peak became broader, in agreement with previous work^14^. The density of adsorbed hydrogen atoms in hydrogenated graphene was estimated from the ratio of peak-height intensities of the D and G bands^40,41^, *I*_D_/*I*_G_. In our devices, *I*_D_/*I*_G_ ≈ 1, which corresponds to a distance between hydrogen atoms in graphene of *L*_D_ ≈ 1 nm. An equivalent analysis using the integrated-area ratio in these peaks^42^, *A*_D_/*A*_G_ ≈ 2, yields *L*_D_ < 1.2 nm. Both estimates yield a density of hydrogen atoms of around 1 × 10^14^ cm^−2^, in agreement with previous reports on hydrogenated graphene^13,14^.

### Electrolyte characterization

To characterize the limiting conductivity of our devices, we measured devices similar to those described above but in which the aperture in the silicon nitride substrate was not covered with graphene (‘open hole device’). Extended Data Fig. 9 shows that the *I*–*V* characteristics of these open-hole devices were linear in all the *V*-bias range used in this work. This demonstrates that the field effect we observed in graphene devices did not arise from changes in the electrolyte conductivity at high *V*, consistent with the known large electrochemical window of this electrolyte (4–5 V; ref. ^43^).

To characterize the capacitance of the electrolyte, we patterned two gold electrodes on a silicon nitride substrate using photolithography and electron-beam evaporation. The electrodes were connected in an electrical circuit and a polymer mask was used to cover all the electrodes, except for an active area that was exposed to the electrolyte. The area of the electrodes differed by a factor of around 50, which ensured that the total capacitance was dominated by the smaller one and allowed us to observe differences, where present, in the response of the devices under positive and negative potentials^44^. Cyclic voltammetry (CV) measurements with scan speeds in the range 1–40 mV s^−1^ were performed over the voltage range −0.1 V to 0.1 V. Extended Data Fig. 6a shows that the CV curves displayed no redox peaks or asymmetry between the positive and negative voltage branches. The area-normalized capacitance of the electrolyte, *C*, could then be obtained from the CV curves from the expression^44,45^ *C* = (*A* × Δ*V* × *ν* )^−1^ ∫ *I* d*V*, where *A* is the active area of the electrode, Δ*V* is the voltage range in the CV, *I* is the current and *v* is the scan speed. Extended Data Fig. 6b shows the extracted *C* as a function of *v*. For the smallest scan rate (1 mV s^−1^), we found *C* ≈ 30 µF cm^−2^. This value decreases with *v* increases, as expected. Because our measurements use *v* = 10 mV s^−1^, we used the value obtained at such *v*, *C* ≈ 20 µF cm^−2^, in our estimates involving *C*.

### Estimation of *E* and *n*

The Debye length in our electrolyte (0.18 M salt and solvent dielectric constant, *ε*_r_ ≈ 10)^46,47^ can be estimated as *λ*_D_ ≈ 0.3 nm. Given this and the relatively large gate bias used in this work, the electrical potential across the graphene–electrolyte interface in our devices dropped almost entirely across the Stern layer. Hence, each of the gate potentials can be described using a parallel plate capacitor model, which we used to derive the relations between the gate potentials and *E* and *n*, as shown in refs. ^25–27,48,49^.

To derive the relation between *V*_t_ + *V*_b_ and *n*, we note that if only one gate (top) operates, the charge induced is *neC*^−1^ = (*V*_t_ − *V*_t_^NP^), where the superscript NP marks the neutrality point and *e* is the elementary charge constant. An equivalent relation holds for the top gate. Hence, the total charge from both gates is given by the addition of their contributions: *neC*^−1^ = (*V*_t_ + *V*_b_) − Δ^NP^, where Δ^NP^ ≡ *V*_t_^NP^ + *V*_b_^NP^. To consider the quantum capacitance of graphene, we note that ${\mu }_{e}={\hbar v}_{{\rm{F}}}\,\sqrt{{\rm{\pi }}n}$, where *v*_F_ ≈ 1 × 10^6^ m s^−1^ is the Fermi velocity in graphene and *ħ* is the reduced Planck constant. This changes the relation to^50^:

1 $$\left({V}_{{\rm{t}}}+{V}_{{\rm{b}}}\right)-{\Delta }^{{\rm{NP}}}={neC}^{-1}+{\hbar v}_{{\rm{F}}}{e}^{-1}{\left(\pi n\right)}^{1/2}$$

From the estimate of *C* above, we get (*V*_t_ + *V*_b_) − Δ^NP^ ≈ (0.8 × 10^−14^ V cm^2^) *n* + (1.16 × 10^−7^ V cm) *n*^1/2^. Note that this description is accurate only if the Fermi energy of the system is outside a bandgap^31^. Hence, we use it only when graphene is conductive. After the hydrogenation transition, we cannot assess *n*, as indicated by the breaks in the top axes in Fig. 2.

To derive the relation between *V*_t_ − *V*_b_ and *E*, we note that if only one gate (top) operates, the electric field induced by the gate is ${E}_{{\rm{t}}}={en}_{{\rm{t}}}{(2\varepsilon )}^{-1}={C(2\varepsilon )}^{-1}({V}_{{\rm{t}}}-{V}_{{\rm{t}}}^{{\rm{NP}}})$, where *ε* is the dielectric constant of the solvent. Note that the electric field points in the direction between graphene and its corresponding electrical double layer, which we define as +*x* for the top gate. An equivalent relation holds for the bottom gate, except that *E*_b_ points in the −*x* direction (towards its corresponding electrical double layer). The total electric field in graphene is then:

2 $$E={E}_{{\rm{t}}}-{E}_{{\rm{b}}}={C(2\varepsilon )}^{-1}\left({V}_{{\rm{t}}}-{V}_{{\rm{b}}}\right).$$

This yields *E* ≈ 1.13 × 10^9^ m^−1^ (*V*_t_ − *V*_b_).

### Analytical model of proton transport and hydrogenation in double-gated graphene

We used an analytical model to illustrate how the gate voltages affect proton transport and hydrogenation in double-gated graphene. The energy barrier for proton transport through the centre of the hexagonal ring in graphene is modelled using a Gaussian function: *V*_p_ = *G*_0_ × exp(−*x*/*W*)^2^, where *G*_0_ = 0.8 eV is the barrier height determined experimentally in the low-electric-field limit^1^ and *W* = 0.5 Å is the barrier width (Extended Data Fig. 7b). For the hydrogenation process, the proton is directed towards the top of a carbon atom in graphene.

The potential energy profile for the hydrogenation process consists of two parts: the energy barrier (*V*_Hb_) and the adsorption well (*V*_Ha_). The energy barrier is modelled with a Lorentzian-type function: *V*_Hb_ = *V*_0_ [((*x* − |*x*_0_|)/*d*_0_)^3^ + 1]^−1^, where *V*_0_ = 0.2 eV is the barrier height, *x*_0_ = 1.7 Å is the distance between the barrier and graphene, and *d*_0_ = 0.4 Å is the barrier width. The third power in the denominator models long-range van der Waals interactions. The adsorption well is modelled with a Lorentzian: *V*_Ha_ = *V*_1_ [((*x* − |*x*_1_|)/*d*_1_)^2^ + 1]^−1^, where *V*_1_ = −0.8 eV is the well depth, *x*_1_ = 1.1 Å is the distance between the well and graphene, and *d*_1_ = 0.25 Å is the width of the well. Note that the well is modelled to be strongly repulsive at *x* = 0 to capture the repulsion between the carbon and hydrogen atoms at very short distances. The parameters for these functions are taken from DFT calculations^14,51^ and the total potential for the hydrogenation process is then *V*_Hb_ + *V*_Ha_ (Extended Data Fig. 7a).

The gate potential profiles (*V*_t_ and *V*_b_) are modelled with a Guoy–Chapman–Stern model^24^, using dielectric constant *ε*_r_ = 10 for the solvent, an electrolyte concentration of 0.18 M and a Stern-layer thickness of 0.4 nm. The resulting gate potentials drop almost exclusively over the Stern layer (Extended Data Fig. 7) and, as a result, the graphene–electrolyte interface behaves as a capacitor, as discussed above. The qualitative findings of our model are relatively insensitive to the specific parameters of the Guoy–Chapman–Stern model if the Stern layer exceeds 0.3 nm. The superposition of the potentials for each of the processes with the gate potentials model the behaviour of the devices.

To illustrate the role of the gates in the hydrogenation process, we set them to yield *n* = 1.2 × 10^14^ cm^−2^ but *E* = 0. Extended Data Fig. 7a shows that this distorts the potential energy profile for hydrogenation, such that the hydrogenation barrier is now easily surmounted by incoming protons, which become trapped in the adsorption well and hydrogenate graphene. To illustrate the role of *E* in the proton-transport process, we set the gates to produce large *E* = 1.7 V nm^−1^ but *n* = 0 cm^−2^. Extended Data Fig. 7b shows that this distorts the potential energy profile for proton transport, such that the barrier is now easily surmounted by a proton moving in the direction of the electric field (from the −*x* to the +*x*). To illustrate the role of electron doping in proton transport, we set the gates to give large *n* = 1 × 10^14^ cm^−2^ but *E* = 0.67 V nm^−1^. Extended Data Fig. 7c shows that this also distorts the potential energy profile for the incoming protons, resulting in facilitated transmission over the barrier. The model illustrates that the distortion of the energy profile for incoming protons due to *E* and *n* in these devices is comparable to the barrier height. For this reason, these variables dominate the response of the devices, and previously identified effects, such as strain and curvature, should have a secondary role.

### Electrochemical description of the hydrogenation process

The transport data are described using the variables *E* and *n*. However, it is equivalent to describe the system using the electrochemical potential of electrons in graphene with respect to the NP, *μ*_e_, instead of *n*. Indeed, one important property of graphene is that *n* and *µ*_e_ are related by the formula ${\mu }_{e}={\hbar v}_{{\rm{F}}}\,\sqrt{{\rm{\pi }}n}$, where *v*_F_ ≈ 1 × 10^6^ m s^−1^ is the Fermi velocity in graphene. This relation is fundamental, arising from the density of states in the material, and holds exactly in experimental systems^52,53^. Moreover, this relation is valid independently of whether the material is gated or not. Hence, the top *x* axis in the hydrogenation map in Fig. 2a can be re-expressed in terms of *µ*_e_, which illustrates that the hydrogenation process is driven by *μ*_e_. This is consistent with the well-established notion that electrochemical charge-transfer processes are driven by this variable. Note that although the relation between *n* and *μ*_e_ is fixed, applying a gate voltage to graphene shifts both variables^31,54^. However, these variables are not independent, as discussed above. To determine their dependence on the gate voltage, we need to establish the electrostatic gate capacitance, *C* (Extended Data Fig. 6). When *C* is determined, the dependence of *n* (or *μ*_e_) on the gate voltage is described by equation (1) above.

### Hydrogenation transition

It is instructive to compare our results with previous work on plasma-hydrogenated graphene^13,55^. In those earlier studies, plasma-hydrogenated graphene typically displayed around 100-times higher electronic resistivity than in the non-hydrogenated state. By contrast, in our work and in ref. ^14^, this factor is about 10^4^, yielding an insulating state that was mostly insensitive to the gate voltage. There are at least two possibilities for this difference. The first is that the hydrogen-atom densities obtained by the different methods are different. Indeed, although the Raman spectra of the current work and ref. ^13^ yield *I*_D_/*I*_G_ ≈ 1, this could arise from a hydrogen-atom density of less than 10^12^ cm^−2^ or around 10^14^ cm^−2^, because of the bell-shape^40,41^ of the graph of *I*_D_/*I*_G_ against defect density. To decide which one applies, it is therefore necessary to look for further evidence of disorder in the spectra. The Raman spectra in ref. ^13^ displayed a sharp 2D band, which is typical of ordered samples and indicates that the hydrogen density was likely to be less than 10^12^ cm^−2^. This contrasts with the 2D band in our spectra, which is smeared, consistent with a figure of around 10^14^ cm^−2^. The second possibility is that both systems had the same hydrogen density. In this case, the higher resistivity could arise from a more disordered hydrogen-atom distribution. Indeed, hydrogen atoms in plasma-hydrogenated graphene are known to cluster^33,56^, which reduces the number of effective scattering centres proportionally to the number of atoms in the cluster. The reduction could be considerable because the scattering radius around each hydrogen atom extends to second neighbours (nine carbon atoms)^33,56,57^. The electrochemical system could be less prone to clusters, perhaps because the electrolyte stabilizes the proton as it adsorbs on graphene, making the reaction more likely to happen than in a vacuum, thus yielding a more random distribution.

Another difference between the two hydrogenation methods is their reversibility. According to ref. ^13^, the plasma-hydrogenation process could be almost completely reversed by annealing the material in an argon atmosphere. However, a D band was still notable after annealing and some of the electronic properties of graphene were not fully recovered^13^. This imperfect reversibility was attributed^13^ to the presence of vacancy defects introduced during the plasma exposure. In both our work and in ref. ^14^, the hydrogenated transition is fully reversible, with no *D* peak apparent in the Raman spectra of dehydrogenated samples.

Another difference with plasma-hydrogenated samples is that electrochemical hydrogenation allows the dependence of the transition on *n* to be studied. This has revealed that the transition is sharp. We attribute this sharpness to a percolation-type transition^58^ triggered both by the high density of adsorbed hydrogen atoms in the samples and to the carrier scattering associated with them^59,60^. We propose that the insulating state in the samples is therefore a consequence of their high disorder, as suggested previously^59,60^, rather than a bandgap. This is consistent with experimental studies reporting that a bandgap in plasma-hydrogenated samples typically requires either the patterned distribution of hydrogen atoms^61^ or a much higher hydrogen-atom density^62^ than in the samples in this work.

### DFT calculations of graphene hydrogenation

Graphene hydrogenation was simulated using the Vienna Ab initio Simulation Package (VASP)^63–66^. Electron–ion interactions were modelled using the projector augmented wave method, and the exchange correlations of electrons were modelled with the Perdew–Burke–Ernzerhof (PBE) generalized gradient approximation functional^67^. Spin polarization was considered and the van der Waals interactions were incorporated by using the Grimme’s DFT-D3 method^68^. Initial crystal-structure relaxation was performed with a force criterion of 0.005 eV Å^−1^ and an electronic convergence of 10^−6^ eV, accelerated with a Gaussian smearing of 0.05 eV. The energy cut-off was set at 500 eV, and Monkhorst–Pack k-point mesh with a reciprocal spacing of 2π × 0.025 Å^−1^ was implemented, which ensured energy convergence to 1 meV. We constructed a cubic simulation, consisting of a 4 × 7 orthogonal supercell with 112 carbon atoms placed at the centre in the *z* direction (perpendicular to the 2D plane) and with a vacuum slab to prevent interactions between adjacent periodic images. After relaxation, the energy barriers for a proton to be adsorbed on top of a carbon atom under vacuum conditions were calculated by ab initio molecular-dynamics simulations using the microcanonical ensemble and the same convergence criteria as mentioned above. We used a time step of 0.1 fs and a minimal initial kinetic energy for the proton in the direction perpendicular to the 2D layer, as previously reported^1,69^. A dipole correction was implemented to study the influence of an external electric field perpendicular to the 2D layer (in the *z* direction)^70^. Owing to the periodic boundary conditions, this dipole is repeatedly inserted in all the simulation boxes in the *z* direction, yielding a constant electric field in the direction perpendicular to graphene^70^.

Extended Data Fig. 8 shows the calculated potential energy curves for the proton–graphene system. The curves were calculated as a function of distance between the proton and the top of a carbon-atom site with a fully relaxed lattice. The potential energy curves display a minimum (adsorption well) at around 1.14 Å (C–H bond) and a small adsorption barrier around 2 Å, in agreement with previous studies^14^. We find the electric field distorts the potential energy profile for hydrogenation, favouring the process in agreement with the analytical model. For reference, we also performed calculations using the non-local optB88-vdW and the hybrid functional HSE06. These resulted only in minor differences (<0.1 eV) in the hydrogenation-barrier height compared with PBE.

### DFT calculations of proton transport through graphene

The DFT calculations of proton transport through graphene were performed using VASP^63–66^ and the plane-wave self-consistent field (PWscf) package with Quantum Espresso (QE). We used the optB88-vdW^71^ functional, with a 3 × 3 × 3 Γ-centred k-point grid, a 1,000 eV energy cut-off with hard pseudopotentials^72,73^, and a force-convergence criterion of 0.03 eV Å^−1^. We used a 4 × 4 unit cell with a vacuum separating periodically repeating graphene sheets of 12 Å for pristine graphene and around 23 Å for hydrogenated graphene. The zero electric field energy profiles were computed using the climbing-image nudged elastic band method^74^ with VASP. Charged cells were used to describe the protons in the simulations with a uniform compensating background. In the model, proton transfer was simulated from a water molecule on one side of graphene to another one on the opposite side. Using these two water molecules minimizes spurious charge transfer from the graphene sheet to the proton, as confirmed with a Bader^75^ charge analysis. To incorporate the electric field, we modelled the system using QE. Here, we used the optB88-vdW functional^71,76–79^, a 3 × 3 × 3 Γ-centred k-point grid and a 600 Ry energy cut-off. We confirmed that the VASP zero electric field energy barriers were reproduced within around 15 meV in QE. The electric field in QE was simulated as a saw-like potential added to the ionic potential, together with a dipole correction implemented according to ref. ^80^. The saw-like potential increased in the region from 0.1 **a**_**3**_ to 0.9 **a**_**3**_, where **a**_**3**_ is the lattice vector perpendicular to the graphene sheet, which was placed at the centre of the cell (0.5 **a**_**3**_), then decreased to 0 at **a**_**3**_ and 0. The discontinuity of the sawtooth potential was placed in the vacuum region. The electric field was applied in the perpendicular direction to the graphene basal plane (the *z* direction). For reference, we also performed calculations using the PBE-D3 functional, which gave comparable results.

We first calculated the energy profile for proton transport through graphene in the absence of an electric field and for two different levels of hydrogen-atom coverage of the lattice (0% and 20%). The choice of 20% hydrogenation was to take into account the fact that adsorbed hydrogen atoms typically form dimer structures consisting of two hydrogen atoms per eight-carbon-atom sublattice^33,56,81^, which correspond to a local lattice coverage of about 25%. In agreement with ref. ^18^, we observed that the energy barrier for pristine graphene reduced by around 30% for 20% hydrogen-atom coverage. The barrier at zero field we found, *Γ*_0_ ≈ 3.1–3.4 eV for the different functionals, is larger than the typically found values^7^ of *Γ*_0_ ≈ 1–2 eV because in our approach the computed proton trajectory involved a chemisorption state, as described previously^18^. However, we note that the absolute values of the barriers in these simplified models are not especially informative, as discussed in ref. ^69^. These models aim to provide only qualitative insights into the influence of *E* and hydrogenation in proton transport through graphene. Next, we computed the energy profiles along the same pathway used in the zero-*E* calculations, but now including a perpendicular electric field, *E*, along the direction of motion of the proton. Extended Data Fig. 10 shows the energy profiles along the reaction path for the two different levels of hydrogenation of the lattice for various electric fields. Regardless of the extent of hydrogenation, we observed a roughly linear barrier reduction when the electric field was switched on, achieving an approximately 20% reduction with *E* at around 1 V nm^−1^.

### Logic and memory measurements

For logic and memory measurements, we defined *V*_t_ and *V*_b_ as the IN1 and IN2 signals, respectively, and, guided by the maps of the devices, we systematically explored their proton and electronic responses to different input signals. To test the stability of the memory states as a function of time, the electronic system was pre-programmed into a conducting (dehydrogenated) or insulating (hydrogenated) state applying *V*_t_ + *V*_b_ = −2.8 V and +2.8 V, respectively. The retention of the insulating state was measured for more than a day with a constant IN1 = IN2 = 0 V, and a reading in-plane *V*_ds_ of 0.5 mV was applied for 20 s every 1,000 s. During logic-and-memory measurements, the electronic system was pre-programmed into a conducting or insulating state as described above. We then applied the input signals. The optimal parameters were found to be 0 V and +1.0 V for both IN1 and IN2 signals, because this yields high *E* but low *n* and thus enables strong modulation of the proton channel with minimum disruption of the electronic memory state. We found that in these measurements, the potentials at which graphene became hydrogenated were larger than in our transport maps. We attribute this to the fact that the fast sweeping of the gates may be altering the composition of the electrochemical double layer, probably resulting in lower concentrations of protons in the graphene–electrolyte interface and thus requiring higher potentials to hydrogenate graphene given the short timescales of this measurement. To implement the logic-and-memory application, the input signals were applied as a function of time in squared waveform patterns. Low and high gate voltages were defined as the logic inputs 0 and 1, respectively, yielding continuous cycles of different input combinations (00, 01, 11, 10).

## Online content

Any methods, additional references, Nature Portfolio reporting summaries, source data, extended data, supplementary information, acknowledgements, peer review information; details of author contributions and competing interests; and statements of data and code availability are available at 10.1038/s41586-024-07435-8.

## Data Availability

The data used in this paper are available from the corresponding authors and at Zenodo at https://zenodo.org/record/10944915 (ref. ^82^).
